# Vision in Film

**DOI:** 10.1371/journal.pbio.0030249

**Published:** 2005-07-12

**Authors:** Sanjit Bagchi

## Abstract

A new film set in India re-creates the story of Helen Keller.

India produces the largest number of movies in the world, and the country is also home to one of the largest number of people born blind [1]. Yet it is only now that the 90-year-old Hindi film industry, based in Mumbai, has made a movie based on the life of Helen Keller, arguably the most inspirational visually challenged person in history.

Because films are supposed to be a means of transient escapism for millions of poor Indians, few movie producers dare to deviate from unrealistic and hackneyed formulae (either fairy-tale romances or action-packed good-versus-evil sagas; in both cases, at least five song-and-dance sequences complete the storyline). With a few exceptions (e.g., *Koshish* and *Sparsh*), rarely have Indian movie-makers dared to foray into more challenging territory.

However, in the past few years, a handful of young directors have begun to venture into important medical territories, such as AIDS *(Phir Milenge)* and autism *(Koi Mil Gaya)*. Sanjay Leela Bhansali has been among the leaders of this nascent movement. After exploring the world of a deaf-mute couple in his debut film five years ago, Bhansali now depicts the indomitable spirit of a deaf, blind, and mute girl in *Black*. More audacious than before, he has added a new twist to the scaffold of the true Helen Keller story, in the form of Alzheimer's disease—a degenerative disorder affecting at least 3 million people in India, yet grossly unrecognised and under-diagnosed due to lack of awareness [2]. The film, poised to be a smash hit, may serve not only to entertain but also to educate.

*Black* is loosely based on *The Miracle Worker*, the Oscar-winning 1962 Hollywood movie about the life of Helen Keller and her teacher, Anne Sullivan. The real Helen, born in 1880 in Tuscumbia, a small rural town in northwest Alabama (United States), lost her sight and hearing to a mysterious disease (probably meningitis or scarlet fever) when she was 19 months old. The otherwise intelligent girl grew up to be a difficult child, only to be sobered by her teacher.

Sullivan began her lessons by teaching Helen to finger spell. Painstakingly, she would spell each word on the girl's wrists as D-O-L-L, C-A-K-E, W-A-T-E-R, and so on, while Helen was made to feel the objects. In the beginning, she could repeat the finger movements on her own but could not understand what they meant. The initial progress was extremely slow because Helen's parents were opposed to the strict discipline Sullivan advocated. Moreover, Sullivan found it extremely difficult to control Helen's temper tantrums and unruly manners.

To improve Helen's behaviour as well as to teach her to communicate, Sullivan moved with her into a small cottage close to the Kellers' home, but away from her parents' compassion. She did not hesitate to punish when necessary, even by refusing to “talk” through finger spelling when the girl threw tantrums. Within a few weeks, Helen's behaviour improved and a bond grew between the two. Eventually, Sullivan taught Helen to not only communicate through sign language but also read Braille books and lip-read through touch. Ultimately, Helen earned a college degree, wrote autobiographical books, including the classic *The Story of My Life*, and toured the world with Anne Sullivan.

In Bhansali's interpretation, Tuscumbia is replaced by Simla, the picturesque hill station in Northern India. Michelle McNally is the deaf-blind girl born to a wealthy Anglo-Indian household. Eight-year-old Ayesha Kapoor brilliantly portrays the frustrations of a child incapable of expressing herself. Unable to see, hear, or talk, Michelle throws violent tantrums, and her parents decide to put her in an asylum. This is when an eccentric teacher, Debraj (played by the Indian idol Amitabh Bachchan), enters her life, taking up the seemingly impossible challenge to illuminate Michelle's “black” existence. Bhansali not only reverses the gender of Helen Keller's real-life teacher but also portrays him as an aging alcoholic and an emotional wreck who has survived a traumatic childhood.

The subplot works well as Debraj, the protagonist, speaks against the ills of institutionalized rehabilitation of the deaf-blind. From the credit lines, it is evident that Bhansali's team has worked closely with members of the Helen Keller Institute of Mumbai for several years. The film drives home the message that even the deaf-blind can lead an independent life with proper care and support. As Debraj says, “Michelle, you've to stand up, or else they'll put a bell on your neck and you'll live like an animal.”

Debraj's strategy for rehabilitation focuses on bringing the experiences of the world to the fingertips of the deaf-blind person. It emphasizes the use of all possible non-linguistic modes of communication, such as gestures, facial expressions, and body movements. The film also portrays rehabilitation of the deaf-blind and mute girl as hard-won and awe-inspiring. It demonstrates that even without modern intervention facilities—such as computerized Braille, talking books, or text readers—that have made rehabilitation of the deaf-blind more systematic and comprehensive since the days of Helen Keller, poor Indian families can offer rich lives to children with such physical and sensory challenges. It encourages parents to create family-centred, home-based programmes, particularly for children who cannot afford to join day-care centres due to lack of suitable transportation or sufficient socio-economic status.

Even more commendable is the film's attempt to introduce Indian viewers to the vagaries of Alzheimer's disease. The audience can readily identify with an ever-enthusiastic and boisterous Debraj, who begins to suffer the abrupt memory lapses that are early signs of the disease. His eyes lose their penetrative look, and he forgets simple words and their connotations. The actor paints the pain of the mind-crippling and devastating illness with great sensitivity.

The film ends with a surrealistic note when Michelle decides to apply the same multi-sensory method on her teacher to lift him from the world of forgetfulness. However, in practical terms, it seems unlikely that rehabilitation originally meant for the deaf-blind and mute will succeed in a patient afflicted with Alzheimer's disease. In fact, the neurological, cognitive, and psychological ravages of the disease in an advanced stage are so destructive that they are completely irreversible.

Despite this unrealistic hope, the film wonderfully drives home the principal message against institution-based therapy; it thoroughly stresses home-based care and empathy. This is particularly important from the Indian viewpoint, for two reasons. First, the country doesn't have enough rehabilitation centres for Alzheimer's disease, even though the number of patients is steadily rising amidst the enormous aging population. Second, the majority of poor Indians will never be able to afford institutionalized care. Proper understanding of the disease and family-centred and home-based interventions, where possible, will be a growing need in India. Films such as *Black* inspire us to rise to the challenge of necessity.
